# Quantitative Detection of *Bifidobacterium longum* Strains in Feces Using Strain-Specific Primers

**DOI:** 10.3390/microorganisms9061159

**Published:** 2021-05-28

**Authors:** Yue Xiao, Chen Wang, Jianxin Zhao, Hao Zhang, Wei Chen, Qixiao Zhai

**Affiliations:** 1 State Key Laboratory of Food Science and Technology, Jiangnan University, Lihu Road No.1800, Binhu District, Wuxi 214122, China; xiaoyuejndx@sina.com (Y.X.); 7180112085@stu.jiangnan.edu.cn (C.W.); zhaojianxin@jiangnan.edu.cn (J.Z.); zhanghao61@jiangnan.edu.cn (H.Z.); chenwei66@jiangnan.edu.cn (W.C.); 2 School of Food Science and Technology, Jiangnan University, Wuxi 214122, China; 3 National Engineering Research Center for Functional Food, Jiangnan University, Wuxi 214122, China; 4 Institute of Food Biotechnology, Jiangnan University, Yangzhou 225004, China; 5 Wuxi Translational Medicine Research Center and Jiangsu Translational Medicine Research, Institute Wuxi Branch, Wuxi 214122, China; 6 Beijing Innovation Centre of Food Nutrition and Human Health, Beijing Technology and Business University (BTBU), Beijing 100048, China; 7 International Joint Research Laboratory for Probiotics at Jiangnan University, Wuxi 214122, China

**Keywords:** strain-specific qualification, probiotics, *B. longum* sup. *longum*, bioinformatics, Roary, gut colonization

## Abstract

We adopted a bioinformatics-based technique to identify strain-specific markers, which were then used to quantify the abundances of three distinct *B. longum* sup. *longum* strains in fecal samples of humans and mice. A pangenome analysis of 205 *B. longum* sup. *longum* genomes revealed the accumulation of considerable strain-specific genes within this species; specifically, 28.7% of the total identified genes were strain-specific. We identified 32, 14, and 49 genes specific to *B. longum* sup. *longum* RG4-1, *B. longum* sup. *longum* M1-20-R01-3, and *B. longum* sup. *longum* FGSZY6M4, respectively. After performing an in silico validation of these strain-specific markers using a nucleotide BLAST against both the *B. longum* sup. *longum* genome database and an NR/NT database, RG4-1_01874 (1331 bp), M1-20-R01-3_00324 (1745 bp), and FGSZY6M4_01477 (1691 bp) were chosen as target genes for strain-specific quantification. The specificities of the qPCR primers were validated against 47 non-target microorganisms and fecal baseline microbiota to ensure that they produced no PCR amplification products. The performance of the qPCR primer-based analysis was further assessed using fecal samples. After oral administration, the target *B. longum* strains appeared to efficiently colonize both the human and mouse guts, with average population levels of >10^8^ CFU/g feces. The bioinformatics pipeline proposed here can be applied to the quantification of various bacterial species.

## 1. Introduction

Intestinal commensals play an important role in host health via being involved in various aspects of host physiology, such as tissue development, metabolism, and immunomodulation [1,2]. Many of these organisms are believed to be beneficial to the host. *Bifidobacterium* is a genus of bacterial species that colonizes the gut early in life [3] and is considered beneficial to host health [4]. The abundances of various *Bifidobacterium* species in the gut vary widely among individuals according to differences in dietary patterns [5,6], age groups [7], and physiological statuses [8]. Among these species, *B. longum* stands out as a member of the core human microbiome [9] and the most dominant species within the *Bifidobacterium* genus in the gut, regardless of the host age [7]. *B. longum* is distributed broadly across subjects of various ages [10], and is among the limited number of bacterial species that can colonize the gut over years [11]. Therefore, *B. longum* is an excellent example of host–microbe co-evolution, and is considered to be among the most potent probiotic species that are likely to engraft and persist in the gut after oral ingestion [12].

Compared with probiotic strains that merely transit through the gut, those probiotic strains that are able to successfully reside in the gut, would interact closely with the gut immune system, mucosa, epithelial cells, and native microbial communities, thereby possibly harboring better probiotic effects. However, as a consequence of the difficulty of strain-level detection, there is clearly a knowledge gap regarding gut colonization mechanisms of probiotics [13]. Current generally used approaches to detect strain colonization include plate counting and species-level PCR [14,15,16]. However, these methods are not accurate, considering the natural occurrence of phylogenetically related species with the ingested probiotic strains in the indigenous microbiota. Therefore, detection and quantitation of probiotics at the strain level are critically important for accessing gut colonization by various strains, and further understanding their functionality and related mechanistic insight.

Multiple approaches have been developed to measure the presence and abundance of specific probiotic strains in the gastrointestinal tract. Initially, methods based on selective culture medium and colony identification (e.g., bacterial morphology, biochemical analysis, pulsed field gel electrophoresis (PFGE), 16S ribosomal DNA (rDNA) PCR, internally transcribed spacer (ITS)-PCR, random amplified polymorphic DNA (RAPD)-PCR, and monoclonal antibodies) were commonly used [14,17,18,19,20,21]. However, these methods are time consuming, laborious, and often inaccurate. Fluorescence [22] or antibiotic labeling [23], and group-specific fluorescence in situ hybridization (FISH) [24] are also ineffective, because of the recurrent loss of plasmids with these tags by strains during gut transition, the low detection sensitivity of fluorescence signals, and safety considerations regarding the application of these approaches in human subjects. Species-specific PCR assays that target 16S rDNA variable regions or 16S-23S ITS rDNA sequences have also been used to directly determine ingested probiotic strains in fecal samples [25,26]. However, this approach cannot distinguish the target strain from phylogenetically related species present in the baseline microbiota. Recently, with the accumulation of sequenced bacterial genomes, strain-specific gene markers have been identified at unprecedented speeds, impelling us to use these unique markers to detect and quantify strains using molecular methods.

Strain-specific detection depends on the identification of DNA regions unique to specific strains. Before the era of large-scale genomic sequencing, selected RAPD electrophoresis bands, specific DNA fragments from suppression subtractive hybridization (SSH), or known sequences related to specific traits (e.g., *Lactobacillus rhamnosus GG* (LGG) harbors a pili structure, but LC705 does not) were used to design strain-specific primers for qualification of some probiotic strains in the gut/fecal samples, including LGG [27], *B. bifidum* OLB6378 [28], *L. gasseri* K7 [29], *B. breve* Yakult [30], and *L. reuteri* DSM 16350 [31]. However, this strain specificity remained within narrow confidence intervals because the identification of the strain-specific DNA regions was based on a limited number of bacterial strains. Additionally, these methods usually required pure cultures of various bacterial strains for laborious electrophoretic analyses. Fortunately, recent emerged bioinformatics strategies based on the sequenced bacterial genomes provide an alternative to find nearly “true” strain-specific DNA sequences. Theoretically, some bioinformatics pipelines (Pan-Seq [32], PGAT [33], PGAP [34], and Roary [35]) can be used to search bacterial strain-specific DNA segments, which can subsequently be used as templates for strain-specific primers. However, no previous study has used these bioinformatics tools to identify strain-specific sequences and achieve strain-level bacterial detection.

In this study, we selected *B. longum* sup. *longum* as an example due to the fact that it has been reported to be the most potent probiotic species with long-term gut colonization potential, and used genomics analyses to identify DNA sequences specific to three *B. longum* sup. *longum* strains isolated from the fecal samples of three Chinese subjects. First, we used a Roary-based pangenome analysis to identify unique gene markers that were present only in a single strains of *B. longum* sup. *longum* but absent from all the other strains of this species. Next, we validated this strain specificity in the context of other microbes and the baseline microbiota, and then targeted these unique sequences to design strain-specific primers. Finally, we applied these strain-specific primers and quantitative PCR (qPCR) to quantify the colonized biomasses of these three *B. longum* sup. *longum* strains in the feces of humans and mice after ingestion.

## 2. Materials and Methods

### 2.1. Bacterial Strains, Culture Conditions and Genomic DNA Extraction

As shown in Table 1, 48 bacterial strains were used in this study. The genomic DNA of each bacterial strain was extracted using rapid bacterial genomic DNA isolation kit (Sangon Biotech Co., Ltd., Shanghai, China).

### 2.2. Bacterial Genome Sequencing and Retrieval of Publicly Available Genomes

Three *B. longum* strains (RG4-1, FGSZY6M4, and M1-20-R01-3) were isolated from fecal samples of three Chinese individuals, and under genome sequencing by Illumina HiSeq 2000. Briefly, a paired-end sequencing library (average insert size of 350 bp and maximum read length of 150 bp) was built according to manufacturers’ instructions (Illumina Inc., San Diego, CA, USA). On average, 3 GB paired-end raw reads were generated for each sample. After removing adaptors and low-quality reads, the resulting clean reads were assembled using SOAPdenovo v2.04 Software for short-read de novo assembler, BGI HK Research Institute: Hong Kong, China, 2012 [36], as described previously [37]. A total of 202 publicly available *B. longum* genomes were downloaded from National Center for Biotechnology Information (NCBI) database (Table 2). In total, 205 *B. longum* assemblies were finally used in this study.

### 2.3. Single Nucleotide Polymorphism (SNP) Calling and Phylogeny Reconstruction

The SNPs were recalled for 205 *B. longum* genomes by mapping the assemblies against the reference genome (*B. longum* NCC 2705) using MUMmer [38], as previously described [37], and only bi-allelic SNPs in the core genome were included in following analysis. The sequences of concatenated SNPs were used to construct phylogenetic tree (neighbor-joining method) using TreeBest (http://treesoft.sourceforge.net/treebest.shtml, accessed on 23 September 2020). It should be mentioned that before conducting the above phylogenetic analysis, we confirmed that the used 205 *B. longum* assemblies belonged to *B. longum* sup. *longum* by building a phylogenetic tree based on core genome bi-SNPs with the genomes of *B. longum* subsp. *suillum*, *B. longum* subsp. *infantis*, and *B. longum* subsp. *suis* as the outgroup.

### 2.4. Identification of Strain-Specific Markers

*B. longum* genomes were re-annotated using Prokka [39], and the obtained protein sequences were used to perform pangenome analysis via Roary (with a minimum BLASTP percentage identity of 90%) [35]. The genes that were only present in a single newly sequenced strain (RG4-1, FGSZY6M4 or M1-20-R01-3) and absent from all the other 204 strains were preliminarily identified. Considering the above gene presence/absence analysis was conducted at the protein level, we further validated the specificity of these strain-specific genes in nucleotide level. A nucleotide database containing 205 *B. longum* genomes was constructed using the makeblastdb command, and the above strain-specific gene sequences were analyzed through BLASTN against this database [40]. The DNA sequences that were only present in the target strain were retained. After the validation for intra-species specificity, we then tested the specificity of the DNA sequences under the background of all the representative microbes via website-based nucleotide BLAST against NR/NT database of NCBI database. The DNA sequences showing no hit in the database were finally selected.

### 2.5. Design of Strain-Specific Primers and Validation of Their Specificity via Electrophoresis

Based on the selected strain-specific DNA sequence for each *B. longum* strain, we designed corresponding qPCR primer pairs using Primer Premier5.0. The specificity of each primer pair was checked by Primer-blast in NCBI database. We conducted three titers of electrophoresis analysis to evaluate the primer specificity. For the intra-species specificity, another ten *B. longum* strains, in addition to each target strain (RG4-1, FGSZY6M4 or M1-20-R01-3), were used. For the intra-genus specificity within *Bifidobacterium*, six other *Bifidobacterium* species were selected. For the specificity against other gut bacteria, 32 representative members of intestinal microbes were adopted. Genome DNA was extracted for each strain, and amplification was performed using the designed strain-specific primers. Bio-Rad T100 Thermal Cycler was used for PCR amplifications. Each reaction mixture (50 µL) consisted of 2 µL bacterial genomic DNA, 25 µL 2× Taq Plus MasterMix, 2 µL forward primer (0.4 µmol in the final mixture), 2 µL reverse primer (0.4 µmol in the final mixture), and 19 µL ddH_2_O. The PCR conditions were as follows: 94 °C for 2 min, followed by 94 °C for 30 s, 65 °C for 30 s, and 72 °C for 30 s conducting 35 cycles, and then 72 °C for 2 min. The electrophoresis analysis was conducted to separate PCR products at 120 V using a 1.5% agarose gel.

### 2.6. Strain-Specific qPCR Designs and Standard Curves for Absolute Quantification

To evaluate the specificity of these strain-specific primer pairs against the background of complex fecal bacterial communities, we collected fecal samples from 30 humans and 15 mice. These samples were all the bassline samples (i.e., before *B. longum* administration) in the following described animal experiment and human trial. The fecal DNA was extracted using FastDNA Spin Kit for Soil (Catalog number: 116570200, MP Biomedicals, Santa Ana, CA, USA) according to the manufacturers’ instructions. The PCR program was optimized to ensure no positive amplification for these baseline samples. Positive control with DNA of each target *B. longum* strain and negative control using water instead of genomic DNA were included in all PCR runs. The PCR system (20 μL) consisted of 2 μL genomic DNA (template), 10 μL 2× supermix (BIO-RAD), 2 µL forward primer (0.4 µmol in the final mixture), 2 µL reverse primer (0.4 µmol in the final mixture), and 4 µL ddH_2_O. The following qPCR program yielded the most selective amplification: an initial denaturation step at 95 °C for 2 min; 30 cycles of denaturation at 95 °C for 5 s and annealing/extension at 65 °C for 30 s; a melt curve analysis between 65 °C and 95 °C in 0.5 °C increments at 2–5 s/step; and polymerase activation and DNA denaturation at 95 °C for 5 min.

For absolute quantification of target *B. longum* strains, standard curves were prepared. A pure culture-based standard series of each target *B. longum* strain was obtained using DNA extracted from a tenfold dilution series of each *B. longum* strain in MRS (16 h). The exact bacterial cell numbers of the first serial decimal dilution were determined using plate counting. Cycle threshold values (C_T_) were plotted versus equivalent log cell numbers. The amplification efficiency of each design was determined by the slope of the standard curves: E (%) = (10^−1/slope^ − 1) × 100.

### 2.7. Quantification of Ingested B. longum Strains in the Fecal Samples

Each *B. longum*-specific qPCR system was used to access the colonized biomass of corresponding target strain in the gut of mice after strain administration. For animal experiments, 5-week-old male Balb/c mice used in this study were purchased from the Shanghai Laboratory Animal Center (Shanghai, China). Animal care and study protocols were approved by the Ethics Committee of Jiangnan University, China (JN. No20181130b1200130[261]). All of the applicable institutional and national guidelines for the care and use of animals were followed. All mice were kept in the mouse facility of the Laboratory Animal Center of the Department of Food Science and Technology, Jiangnan University, Wuxi, China, on a 12-h light/dark cycle in a temperature-(22 °C ± 1 °C) and humidity-controlled (55% ± 10%) room. Mice were assigned to three experiment groups (*n* = 5): the RG4-1 group, the FGSZY6M4 group and the M1-20-R01-3 group. The experimental period was 14 days, including a 7-day accommodation period, and followed by a 7-day *B. longum* intervention period. MRS broth-cultured *B. longum* strains, after being resuspended in sterile saline, were prepared each day, and routinely subjected to plate counting to ensure a gavage dose of 10^8^–10^9^ CFU/d for each mouse. Fecal samples were collected at day 7 and day 14.

For the human trial, the 30 volunteers were students at Jiangnan University who ranged in age from 20 to 30 years. No use of antibiotics was reported by the subjects within the 1 month before or during the study, and probiotic foods were not allowed during the trial. The experimental period included a 2-week baseline period (without any treatment) and a 2-week probiotic intervention period. The subjects were randomly assigned to three groups (*n* = 10 for each group), in which each subject in each *B. longum* intervention group (RG4-1, FGSZY6M4 or M1-20-R01-3) was administrated 10^9^–10^10^ viable cells/d of the corresponding *B. longum* strain. Fecal samples were collected at day 14 (±3 days) of the baseline period, and day 14 (±3 days) of the treatment period. The colonized biomass of the three *B. longum* strains in fecal samples was determined using the respective qPCR primers. All volunteers provided informed consent. The Ethics Committee of Jiangnan University (Wuxi, China) provided ethical clearance for this human trial in accordance with the Declaration of Helsinki.

The colonized biomass of each *B. longum* strains was confirmed by selective culture medium with colony typing with the designed strain-specific primers. In brief, fecal samples were used to isolate bifidobacteria by cultivation on deMan Rogosa Sharpe (MRS) agar supplemented with 50 mg/L mupirocin and 0.1% L-cysteine HCl. After incubation at 37 °C for 48 h in an anaerobic chamber (80% N_2_, 10% H_2_, 10% CO_2_), colonies were counted, picked, and then subjected to conventional PCR using the strain-specific primers.

## 3. Results

### 3.1. Genomic Diversity of B. longum

**Figure 1:**
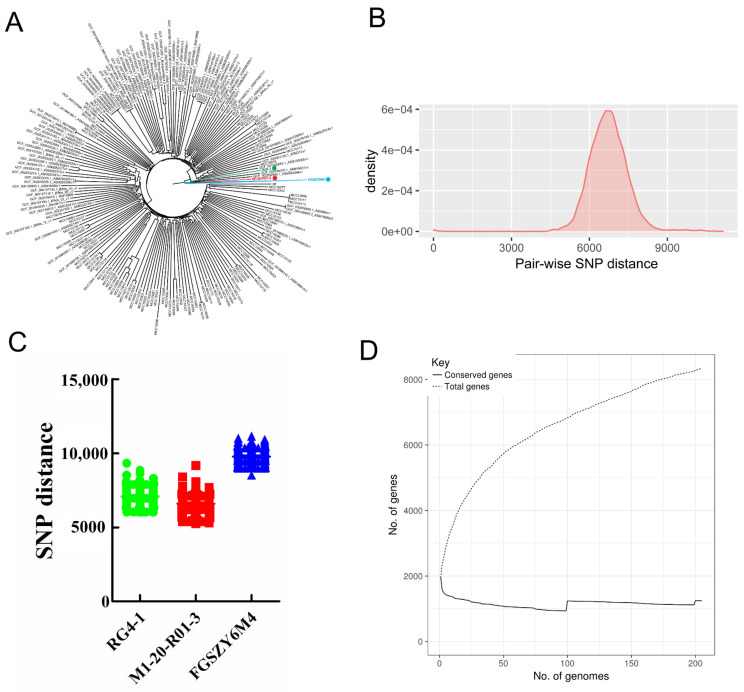
Phylogenetic relationship and genomic diversity of *B. longum*. (**A**) Phylogenetic tree (neighbor-joining method) of 205 *B. longum* strains. The three target strains used for strain-specific detection are colored. (**B**) Distribution of pair-wise SNP distances between 205 strains. (**C**) Pair-wise SNP distances between each of the target strains and all the other strains in the dataset. (**D**) Pangenome curve depicting the number of total genes detected versus the number of conserved genes as the number of included genomes increases.

We reconstructed a phylogenetic tree based on three newly sequenced and 202 publicly available *B. longum* strain genomes (Table 2 and Table 3, and Figure 1A), and calculated the pair-wise genetic distances and accessory gene numbers to reveal the intra-species genomic diversity (Figure 1B–D). The parameters for the newly sequenced genomes are shown in Table 3. As shown in Figure 1A, the three *B. longum* strains (RG4-1, M1-20-R01-3, and FGSZY6M4) isolated from the fecal samples of three Chinese individuals are closely clustered in the phylogenetic tree. This is unsurprising, because the majority of publicly available strains were isolated from geographically distant areas (i.e., other countries or continents). Nevertheless, the three strains exhibited obvious genetic distances indicative of their distinct genotypes.

We also determined the number of variable SNPs in the core genomes of 205 *B. longum* strains (Figure 1B). The results indicated that the average SNP distance across the 205 strains was 6691. The most phylogenetically related strains exhibited an SNP distance of 0, while the most phylogenetically distant strains exhibited an SNP distance of 11,145. For each of the three novel *B. longum* strains, the minimum genetic distances between the respective strain and the other 204 strains in the dataset were 6000 (RG4-1), 5257 (M1-20-R01-3), and 8514 (FGSZY6M4). These data further confirm the genetic differences between each of these three *B. longum* strains and the other strains (Figure 1C). As shown in Figure 1D, the pangenome analysis indicated that accessory genes accounted for 85.1% of the total genes, and that new genes were accumulated frequently as new strains were added to the analysis.

Overall, *B. longum* showed a high level of intra-species genomic diversity in terms of the pair-wise SNP distances and the total numbers of accessory genes. The target strains selected for strain-specific detection were phylogenetically distinct and exhibited considerable genetic dissimilarity with the other strains in this dataset, thus implying the possibility of finding appropriate strain-specific markers.

### 3.2. In Silico Identification and Validation of Strain-Specific Gene Markers

**Figure 2:**
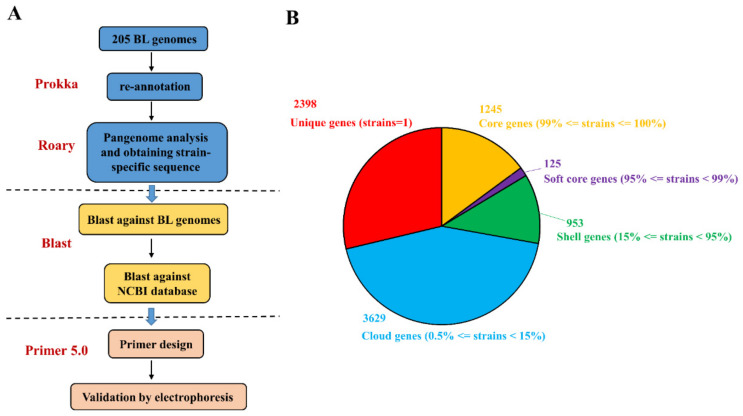
The analysis pipeline for strain-specific primer design corresponding to the three *B. longum* strains (**A**) and the pangenome readout (**B**).

Considering the relatively high intra-species genetic similarity relative to inter-species and inter-genus similarities, we initially searched for strain-specific genes within the *B. longum* genomes. The pipeline used to construct a strain-specific detection tool is shown in Figure 2A. The publicly available *B. longum* genomes used in this study were derived from different projects, and the formats of their annotation files were not uniform, which prevented a pangenome analysis. Accordingly, we re-annotated these publicly available genomes and the three target *B. longum* strain genomes using Prokka, and then conducted a gene presence/absence analysis based on the annotated protein sequences. We defined unique genes (present in only one strain within the dataset of 205 strains), core genes (present in ≥99% of strains), soft core genes (present in ≥95% to <99%), shell genes (present in ≥15% to <95%), and cloud genes (present in ≥0.5% to <15%). As shown in Figure 2B, this analysis preliminarily identified 2398 strain-specific genes across the 205 *B. longum* strains, which accounted for 28.7% of the total genes. Notably, 32, 14, and 49 strain-specific genes were identified, respectively, for *B. longum* RG4-1 (Table 4), *B. longum* M1-20-R01-3 (Table 5), and *B. longum* FGSZY6M4 (Table 6).

Next, we explored whether the specificities of the identified strain-specific genes would be maintained against other microbial taxa. Rather than using amino acid sequences in Roary analysis, we tested the specificities of the preliminary strain-specific genes at the nucleotide level using an in-house nucleotide BLAST against a *B. longum* genome database to further ensure the strain-specificity of the identified genes. We also used a website-based nucleotide BLAST tool against the NR/NT database (NCBI) to determine which sequences could not be identified in any other representative microbes. Finally, this screening process identified RG4-1_01874 (1331 bp), M1-20-R01-3_00324 (1745 bp), and FGSZY6M4_01477 (1691 bp), which were selected as the target DNA sequences for strain-specific quantification.

### 3.3. Strain-Specific qPCR Designs and Electrophoretic Validation

**Figure 3:**
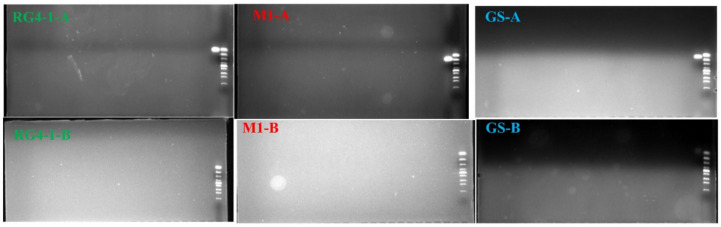
Electrophoresis results of PCR products generated using each strain-specific qPCR primer pair against DNA from the respective target *B. longum* strains and non-target microorganisms. Each gel includes 25 lanes (including a lane for the gene ruler). Order of microorganisms were as follows: for RG4-1-A (from right to left), gene ruler, *B. longum* RG4-1, *B. longum* FGSZY6M4, *B. longum* M1-20-R01-3, *B. longum* 274, *B. longum* FSHHK13M1, *B. longum* FSDLZ57M1, *B. longum* NaTon 49-4, *B. longum* FJSWXJ11M1, *B. longum* HUB 36-17, *B. longum* 28-10, *B. longum* ZCC7, *Bifidobacterium breve* DSM 20213, *Bifidobacterium bifidum* DSM 20456, *Bifidobacterium pseudocatenulatum* FQHXN5M4, *Bifidobacterium pseudolongum* 56M2, *Bifidobacterium animalis* BB12, *Bifidobacterium adolescentis* L2-32, *Lactobacillus salivarius* DSM 20555, *Lactobacillus gasseri* DSM 20243, *Lactobacillus casei* DSM 20011, *Lactobacillus acidophilus* DSM 20079, *Lactobacillus plantarum* DSM 20174, *Lactobacillus reuteri* DSM 20016, and *Lactobacillus rhamnosus* LMS2-1; for M1-A (from right to left), gene ruler, *B. longum* M1-20-R01-3, *B. longum* RG4-1, *B. longum* FGSZY6M4, and the order of following strains was the same as that of RG4-1-A; for GS-A (from right to left), gene ruler, *B. longum* FGSZY6M4, *B. longum* M1-20-R01-3, *B. longum* RG4-1, and the order of following strains was the same as that of RG4-1-A; for RG4-1-B, M1-B and GS-B (from right to left), *Escherichia coli* CMCC44102, *Akkermansia muciniphila* FJLHD50M21, *Faecalibacterium prausnitzii* ATCC 27768, *Enterococcus faecalis* CCFM596, *Bacteroides fragilis* NCTC9343, *Bacteroides thetaiotaomicron* FNMHLBE9-K-7, *Bacteroides eggerthii* FSDTA-HCK-B-9, *Bacteroides cellulosilyticus* FSDTA-ELI-BHI-5, *Bacteroides nordii* FNMHLBE13K2, *Bacteroides stercoris* FJSWX62K34, *Bacteroides uniformis* FJSWX62K43, *Bacteroides caccae* FFJLY22K5, *Parabacteroides distasonis* FSDTA-HCM-XY-12, *Bacteroides dorei* FJSWX61E4, *Bacteroides faecis* FTJS2E2, *Bacteroides intestinalis* FBJ60K5, *Bacteroides vulgatus* FSDLZ51K1, *Bacteroides finegoldii* FNMHLBE11E1, *Bacteroides ovatus* FBJ10-K-10, *Bacteroides clarus* F-FJ-LY 22-K-22, *Bacteroides salyersiae* FSDTA-ELI-BHI-9, *Bacteroides xylanisolvens* FSDTAHCMXY17, *Parabacteroides merdae* FSDTAELIBHI4 and *Clostridium butyricum* FJSCZD1G10.

Next, qPCR primer pairs were designed for each of the three *B. longum* strains (RG4-1, M1-20-R01-3, and FGSZY6M4) based on their respective strain-specific DNA sequences (Table 7), with predicted product sizes of 115, 199, and 144 bp, respectively. Using electrophoresis, the specificities of the three qPCR probe sets were validated against genomic DNA derived from various microbial strains (Table 1), including 10 other *B. longum* strains, 6 other *Bifidobacterium* species, and 32 representative members of the intestinal microbiome. The results indicated that PCR amplification based on the strain-specific primers was only successful for each of the respective target strains, and no amplification of genomic material from non-target microorganisms was observed (Figure 3). Multiple qPCR primer pairs were designed for each target strain, and the primers with the best performance at this stage were selected and are reported here.

### 3.4. Specificities, Standard Curves, and Amplification Efficiencies of qPCR Assays

**Figure 4:**
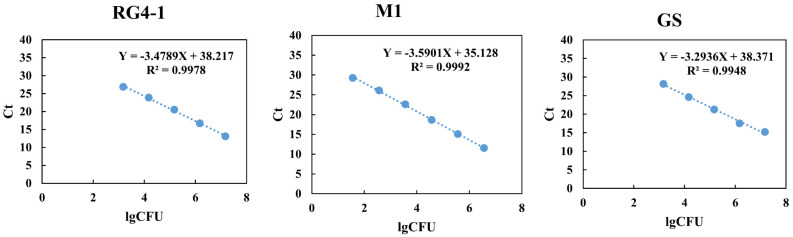
qPCR standard curves for the three *B. longum* strains.

Primer specificity was evaluated by qPCR against a complex microbial community present in baseline fecal samples that had not been enriched for the target strain (i.e., pre-treatment). The DNA of each target *B. longum* strain was used as a positive control in the qPCR runs. Under optimized conditions, no amplification was observed for the baseline samples, whereas the positive controls exhibited good amplification. As shown in Figure 4, standard curves corresponding to RG4-1, M1-20-R01-3, and FGSZY6M4 exhibited good linearity over a 4-log range (10^3^–10^7^ CFU/qPCR system, R^2^ > 0.99), a 5-log range (10^1^–10^6^ CFU/qPCR system, R^2^ > 0.99) and a 4-log range (10^3^–10^7^ CFU/qPCR system, R^2^ > 0.99), respectively. The equations of the regression curves for RG4-1, M1-20-R01-3, and FGSZY6M4 were as follows: Ct = −3.4789lgCFU + 38.217, Ct = −3.5901lgCFU + 35.128, and Ct = −3.2936lgCFU + 38.371; and the corresponding amplification efficiencies were 93.8%, 90.0% and 101.2%, respectively. Collectively, the three qPCR primer pairs exhibited specificity in the context of the complex bacterial communities in fecal samples from both humans and mice, the standard curves used for absolute quantification were of good linearity, and the amplification efficiencies were qualified, suggesting that these primers can be used to detect the abundances of the target *B. longum* strains in fecal samples.

### 3.5. Quantification of Ingested B. longum Strains in Mouse and Human Fecal Samples

**Figure 5:**
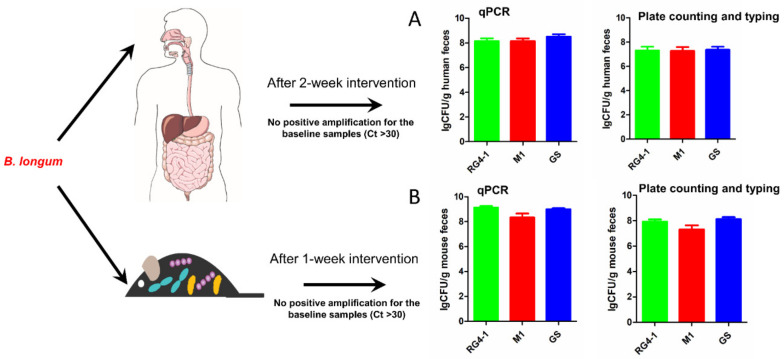
Colonized biomasses of the target *B. longum* strains in fecal samples from humans and mice. Panel (**A**), the results collected from the human trail; Panel (**B**), the results collected from the animal experiments.

We used the strain-specific primers to detect the abundances of three target *B. longum* strains in the feces of mice and human volunteers who had consumed these *B. longum* strains (Figure 5). For the animal experiments, the colonized biomasses of the three *B. longum* strains in samples collected 1 week after oral administration exceeded 10^8^ CFU/g feces, with average abundances of 1.54 × 10^9^ CFU/g feces for RG4-1, 4.60 × 10^8^ CFU/g feces for M1-20-R01-3, and 1.06 × 10^9^ CFU/g feces for FGSZY6M4. For the human trial, the average population levels of each strain reached >10^8^ CFU/g feces, with average abundances of 4.00 × 10^8^ CFU/g feces for RG4-1, 3.78 × 10^8^ CFU/g feces for M1-20-R01-3, and 7.18 × 10^8^ CFU/g feces for FGSZY6M4. These results indicate that orally ingested *B. longum* strains can be detected at considerable levels in the feces of both mice and humans during a period of intervention, suggesting short-term engraftment of the ingested bacteria in the gut. The colonized abundances of the three *B. longum* strains obtained from selective culture medium combined with colony typing using our specific qPCR primers further confirmed the conclusion of short-term engraftment by those strains, despite the bacterial numbers detected by this culture-dependent method were generally 10-fold lower (Figure 5).

## 4. Discussion

It has become increasingly clear that the beneficial effects of probiotic bacteria on the host are strain-specific, and the viability of probiotic strains in the gut after ingestion is believed to be an essential factor for them to demonstrate health-promoting functions. Therefore, determining the presence and colonized biomasses of specific probiotic strains in the gut is a key step toward the informed use of probiotics for therapeutic ends. Compared with traditional microbiological methods (e.g., selective media with colony identification), PCR-based molecular methods, which can distinguish the target strain from the baseline microbiota, are the most popular because of their superior sensitivity and specificity [13]. Here, we adopted a pangenome analysis-based approach to identify strain-specific DNA sequences and designed qPCR primers based on these unique markers, which enabled us to detect *B. longum* strains in fecal samples at a strain-level resolution. In addition to determining the abundances of probiotic bacteria at the strain level, this strategy advantageously involves the identification of strain-specific sequences based on a large set of bacterial genomes. The resulting strain-specificity was more “true” than those identified in an RAPD analysis based on a restricted number of pure cultured bacteria.

This is the first known example of using a bioinformatics method to search for unique gene markers and design strain-specific molecular tools to detect and quantify individual bacterial strains. Previous studies have used the abovementioned RAPD methods to identify strain-specific sequences against a background of a limited number of strains of the same species. Daranas et al. screened a unique marker of *L. plantarum* PM411 against seven other *L. plantarum* strains in an assay based on seven random RAPD primers [41]. In another study, *B. bifidum* BF-1 was detected by targeting a strain-specific sequence in an analysis involving 27 RAPD primers and 30 *B. bifidum* strains [42]. In this study, we identified 32, 14, and 49 strain-specific genes corresponding to *B. longum* RG4-1 (Table 5), *B. longum* M1-20-R01-3 (Table 6), and *B. longum* FGSZY6M4, respectively (Table 7), in the context of 205 *B. longum* genomes. Obviously, our pangenome-based approach could identify a larger number of strain-specific fragments within a wider confidence interval, because it uses whole genome sequences rather than random PCR products, and a large set of bacterial genomes rather than a limited number of available pure cultures. In addition, we also observed a high frequency of strain-specific sequences among *B. longum* isolates. Specifically, we detected 2398 strain-specific genes among 205 *B. longum* strains, which accounted for 28.7% of the total genes, and 178 of these strains harbored unique gene markers. Our data suggest the existence of considerable intra-species genomic diversity within *B. longum* in terms of the accessory gene number. This finding allowed us to construct strain-specific primers to identify and quantify most of these strains based on single gene markers. For those strains without unique genes, future approaches may use two or more gene sequences as unique combination amplification targets.

The strain-specific sequences identified in this study were first confirmed in silico, by electrophoresis and by the absence of qPCR amplification signals in baseline microbiota of the tested fecal samples derived from humans and mice. In previous studies, strain-specific sequences identified using RAPD methods often showed homology with sequences from other microbes and thus were not truly strain-specific. A previous BLAST analysis of a *B. bifidum* OLB6378-specific RAPD fragment showed a high similarity (98%) to the *parB* gene sequences in publicly available *B. bifidum* genomes [28]. The strain-specific RAPD band identified by Karjalainen et al., and used for the strain-level detection of *Propionibacterium freudenreichii* (*P. freudenreichii*). JS was found to encode a 103-bp region with 91% identity to *P. freudenreichii* ssp. *shermanii* CIRM-BIA1 [43]. Similarly, the unique RAPD band for *B. breve* 99 was shown to contain regions homologous to those detected in strains of *B. longum*, *B. adolescentis*, *B. dentium* Bd1, and *B. animalis* ssp. *lactis* [43]. In this study, we validated the strain-specific sequences identified preliminarily via nucleotide BLAST against a self-constructed database of the 205 *B. longum* genomes and further checked the specificities of the sequences against all available gene information from representative microbes via searching the NR/NT NCBI database. Our selected strain-specific fragments (RG4-1_01874, M1-20-R01-3_00324, and FGSZY6M4_01477) passed the nucleotide BLAST against the *B. longum* genome database and generated no hits in the NR/NT database. Therefore, the strain-specific sequences identified in this study were highly specific against a background of various microbes and can probably be applied in a broader taxonomic context. We further validated the specificities of the newly designed qPCR primers via conventional PCR against 10 other *B. longum* strains, 6 other *Bifidobacterium* species, and 32 representative members of the intestinal microbiome. We additionally confirmed the specificities of the primers by the absence of qPCR amplification products from fecal samples derived from both humans and mice that were free from the target *B. longum* strains.

The bioinformatics pipeline proposed in this study can be expanded to search for strain-specific markers and achieve strain-level qualifications of various bacteria, including probiotic species. A large number of genomes corresponding to probiotic *Lactobacillus* and *Bifidobacterium* species have been sequenced and made publicly available, including 544 *L. plantarum*, 198 *L. paracasei*, 112 *B. breve*, and 176 *L. salivarius* genomes in the NCBI database. Previous genomic analyses of these species have demonstrated open pangenomes, high intra-species genomic diversity, and high new gene discovery rates for *L. salivarius* [44,45] and *L. casei* [46] as the number of included genomes increases. Therefore, the pipeline constructed here can be directly translated for use in other probiotic species, provided that sufficient genomic data and abundant strain-specific genes are available. For this study, we selected Roary because of the simple command line and relatively higher running speed compared with other available tools. Other pangenome analysis tools, such as Pan-Seq [32], PGAT [33], and PGAP [34], are also suitable choices for constructing strain-specific primers.

Our qPCR assays further indicated that the three target *B. longum* strains could colonize the guts of both humans and mice at high levels of abundance (>10^8^ CFU/g feces for both humans and mice) during an intervention period (1 week for mice and 2 weeks for humans), and the colonized biomasses were validated using selective culture and colony-typing methods with the strain-specific primers. Absolute qualification was achieved using highly linear standard curves (R^2^ > 0.99) and amplification efficiency was qualified (>90.0%), both of which are comparable to previous studies [41]. In line with our results, a previous study demonstrated that at 2 weeks post-ingestion, the abundances of *B. longum* AH1206 ranged from 10^7^ to 10^10^ cells/g feces in human subjects [47]. Selective culture methods combined with strain typing using our strain-specific primers confirmed the colonization of these target *B. longum* strains. However, the bacterial numbers generated using this culture-based method were nearly 10-fold lower than those determined using qPCR. We attribute this underestimation to an inherent defect of culture-based methods, as previous studies have reported that both the frequencies and numbers of bacterial cells detected in feces by culture methods were substantially lower than the corresponding values obtained using qPCR [42,48]. In addition, PCR with our strain-specific primers enabled us to identify colonies of the three *B. longum* strains on selective agar efficiently and accurately.

## 5. Conclusions

In conclusion, benefiting from the large number of sequenced genomes of probiotic species, we took the most potent probiotic gut colonizer, *B. longum*, as an example, and proposed a precedent in which a pangenome analysis-based approach can be used to identify unique gene markers for a given bacterial strain, and targeted these markers to achieve strain-level qualification. The qPCR primers designed in this study were able to successfully detect and quantify the colonized biomasses of the given *B. longum* strains in fecal samples from humans and mice. Therefore, we demonstrated the ability of these efficient in silico analyses to replace existing time- and labor-intensive RAPD methods. Furthermore, by including as many bacterial genomes as possible, the annotated unique sequences are highly specific and can be applied in a broader taxonomic context involving a more complex microbial ecology. The pipeline constructed herein can also be adapted to identify strain-specific markers and design strain-level qPCR primers for other probiotic species.

## Figures and Tables

**Table 1:** Bacterial strains used for primer validation via electrophoresis ^a^.

Species	Accession Number	Culture Conditions
***Bifidobacterium***		deMan Rogosa Sharpe (MRS) broth supplemented with 0.1% L-cysteine HCl at 37 °C
*B. longum*	RG4-1 ^b^, FGSZY6M4 ^b^, M1-20-R01-3 ^b^, 274 ^b^, FSHHK13M1 ^b^, FSDLZ57M1 ^b^, NaTon 49-4 ^b^, FJSWXJ11M1 ^b^, HUB 36-17 ^b^, 28-10 ^b^, ZCC7 ^b^	
*B.* *breve*	DSM 20213 ^c^	
*B. bifidum*	DSM 20456 ^c^	
*B. pseudocatenulatum*	FQHXN5M4 ^b^	
*B. pseudolongum*	56M2 ^b^	
*B. animalis*	BB12^d^	
*B. adolescentis*	L2-32 ^e^	
***Lactobacillus***		MRS broth at 37 °C
*L. salivarius*	DSM 20555 ^c^	
*L. gasseri*	DSM 20243 ^c^	
*L. casei*	DSM 20011 ^c^	
*L. acidophilus*	DSM 20079 ^c^	
*L. plantarum*	DSM 20174 ^c^	
*L. reuteri*	DSM 20016 ^c^	
*L. rhamnosus*	LMS2-1 ^e^	
**Non-lactic acid bacteria (LAB)**		
*Escherichia coli*	CMCC 44102 ^f^	Luria-Bertani (LB) broth at 37 °C
*Akkermansia muciniphila*	FJLHD50M21 ^b^	Brain Heart Infusion (BHI) broth at 37 °C
*Faecalibacterium prausnitzii*	DSM 17677 ^c^	BHI broth containing 3.7% BHI powder supplemented with 0.5% yeast extract, 0.0005% hemin, 0.0005% vitamin K and 0.2% L-cysteine HCl at 37 °C
*Enterococcus faecalis*	CCFM596 ^b^	BHI broth at 37 °C
*Bacteroides fragilis*	ATCC 25285/NCTC 9343 ^g^	BHI broth supplemented with 0.1% L-cysteine HCl, 0.001% hemin and 0.0002% vitamin K at 37 °C
*Bacteroides thetaiotaomicron*	FNMHLBE9-K-7 ^b^	
*Bacteroides eggerthii*	FSDTA-HCK-B-9 ^b^	
*Bacteroides cellulosilyticus*	FSDTA-ELI-BHI-5 ^b^	
*Bacteroides nordii*	FNMHLBE13K2 ^b^	
*Bacteroides stercoris*	FJSWX62K34 ^b^	
*Bacteroides uniformis*	FJSWX62K43 ^b^	
*Bacteroides caccae*	FFJLY22K5 ^b^	
*Parabacteroides distasonis*	FSDTA-HCM-XY-12 ^b^	
*Bacteroides dorei*	FJSWX61E4 ^b^	
*Bacteroides faecis*	FTJS2E2 ^b^	
*Bacteroides intestinalis*	FBJ60K5 ^b^	
*Bacteroides vulgatus*	FSDLZ51K1 ^b^	
*Bacteroides finegoldii*	FNMHLBE11E1 ^b^	
*Bacteroides ovatus*	FBJ10-K-10 ^b^	
*Bacteroides clarus*	F-FJ-LY 22-K-22 ^b^	
*Bacteroides salyersiae*	FSDTA-ELI-BHI-9 ^b^	
*Bacteroides xylanisolvens*	FSDTAHCMXY17 ^b^	
*Parabacteroides merdae*	FSDTAELIBHI4 ^b^	
*Clostridium butyricum*	FJSCZD1G10 ^b^	Reinforced Clostridial Medium (RCM) at 37 °C

^a^ Anaerobes (*Bifidobacterium*, *Akkermansia muciniphila, Faecalibacterium prausnitzii*, *Bacteroides* strains and *Clostridium butyricum*) were maintained in anaerobic chamber (80% N_2_, 10% H_2_, 10% CO_2_) during cultivation. ^b^ These strains were retrieved from Culture Collection of Food Microorganisms, Jiangnan university. ^c^ These strains were purchased from Deutsche Sammlung von Mikroorganismen und Zellkulturen (DSMZ). ^d^ The strain was isolated from the commercial probiotic product. ^e^ The strains were kindly provided by Biodefense and Emerging Infections Research Resources Repository (BEI Resources). ^f^ The strain was purchased from National Center for Medical Culture Collections (CMCC). ^g^ The strain was purchased from American Type Culture Collection (ATCC).

**Table 2:** Publicly available *B. longum* genomes used in this study.

Genome	Strain	BioSample	Size (Mb)	GC%	Scaffolds	CDS
GCA_001576955.1_ASM157695v1	121.2	SAMN04497913	1.87256	60.3	234	1453
GCA_002331305.1_ASM233130v1	UBA2088	SAMN06457477	1.87849	59.2	227	0
GCF_900157195.1_Bifido_02_v1	Bifido_02	SAMEA51816418	2.33429	60.1	97	1860
GCF_900157165.1_Bifido_12_v1	Bifido_12	SAMEA51823918	2.07288	60.5	681	1743
GCF_900157155.1_Bifido_06_v1	Bifido_06	SAMEA51819418	2.42182	60	48	1978
GCF_900157145.1_Bifido_03_v1	Bifido_03	SAMEA51817168	2.41363	60.1	82	1962
GCF_900157115.1_Bifido_05_v1	Bifido_05	SAMEA51818668	2.33474	59.9	88	1906
GCF_900157095.1_Bifido_01_v1	Bifido_01	SAMEA51815668	2.33463	59.9	39	1907
GCF_900157055.1_Bifido_09_v1	Bifido_09	SAMEA51821668	2.66124	59.9	68	2225
GCF_900104835.1_IMG-taxon_2634166334_annotated_assembly	DSM 20219	SAMN04489748	2.44902	60.3	6	1942
GCF_004334865.1_ASM433486v1	MCC10119	SAMN06368669	2.48689	60.1	45	2023
GCF_004334855.1_ASM433485v1	MCC10122	SAMN06368672	2.46043	60.1	49	1978
GCF_004334815.1_ASM433481v1	MCC10123	SAMN06368673	2.5065	59.7	49	2060
GCF_004334795.1_ASM433479v1	MCC10125	SAMN06368675	2.44774	60.2	43	1979
GCF_004334785.1_ASM433478v1	MCC10128	SAMN06368678	2.50961	59.9	57	2089
GCF_004334775.1_ASM433477v1	MCC10129	SAMN06368679	2.27353	60.1	18	1812
GCF_004334745.1_ASM433474v1	MCC10117	SAMN06368667	2.30167	59.9	35	1807
GCF_004334715.1_ASM433471v1	MCC10120	SAMN06368670	2.48377	60.2	62	2014
GCF_004334705.1_ASM433470v1	MCC10118	SAMN06368668	2.34975	59.9	36	1897
GCF_004334695.1_ASM433469v1	MCC10121	SAMN06368671	2.38447	60	26	1916
GCF_004334645.1_ASM433464v1	MCC10124	SAMN06368674	2.45702	60.1	47	1985
GCF_004334635.1_ASM433463v1	MCC10126	SAMN06368676	2.55307	59.8	68	2055
GCF_004334625.1_ASM433462v1	MCC10130	SAMN06368680	2.36857	60	59	1908
GCF_004334615.1_ASM433461v1	MCC10127	SAMN06368677	2.35673	60.1	41	1874
GCF_004334555.1_ASM433455v1	MCC10212	SAMN06368681	2.36547	59.9	28	1904
GCF_004334545.1_ASM433454v1	MCC10002	SAMN06368569	2.63451	60	59	2202
GCF_004334535.1_ASM433453v1	MCC10006	SAMN06368572	2.45014	60.4	83	1991
GCF_004334515.1_ASM433451v1	MCC10009	SAMN06368575	2.5237	60.1	60	2035
GCF_004334485.1_ASM433448v1	MCC10011	SAMN06368577	2.39746	59.9	34	1931
GCF_004334465.1_ASM433446v1	MCC10016	SAMN06368581	2.37118	60	66	1893
GCF_004334445.1_ASM433444v1	MCC10027	SAMN06368589	2.50583	59.9	59	2042
GCF_004334435.1_ASM433443v1	MCC10019	SAMN06368584	2.30453	60.1	41	1837
GCF_004334425.1_ASM433442v1	MCC10028	SAMN06368590	2.42541	60.2	44	1983
GCF_004334365.1_ASM433436v1	MCC10038	SAMN06368598	2.36701	59.9	38	1968
GCF_004334355.1_ASM433435v1	MCC10030	SAMN06368592	2.51844	60.1	64	2023
GCF_004334345.1_ASM433434v1	MCC10040	SAMN06368600	2.44996	60.2	45	1996
GCF_004334335.1_ASM433433v1	MCC10039	SAMN06368599	2.38905	60	34	1924
GCF_004334325.1_ASM433432v1	MCC10047	SAMN06368607	2.36451	59.9	64	1861
GCF_004334285.1_ASM433428v1	MCC10051	SAMN06368610	2.30363	60.1	42	1830
GCF_004334255.1_ASM433425v1	MCC10057	SAMN06368616	2.18862	59.9	73	1725
GCF_004334245.1_ASM433424v1	MCC10054	SAMN06368613	2.29564	60	61	1842
GCF_004334235.1_ASM433423v1	MCC10059	SAMN06368618	2.43718	60	47	2005
GCF_004334215.1_ASM433421v1	MCC10058	SAMN06368617	2.32956	60.2	65	1886
GCF_004334205.1_ASM433420v1	MCC10072	SAMN06368628	2.23388	60	23	1742
GCF_004334165.1_ASM433416v1	MCC10074	SAMN06368630	2.41001	59.8	34	1965
GCF_004334155.1_ASM433415v1	MCC10077	SAMN06368633	2.41162	60	40	1957
GCF_004334145.1_ASM433414v1	MCC10083	SAMN06368638	2.47557	60.2	64	1953
GCF_004334105.1_ASM433410v1	MCC10085	SAMN06368640	2.3663	60	64	1908
GCF_004334075.1_ASM433407v1	MCC10003	SAMN06368570	2.52677	60.1	69	2047
GCF_004334065.1_ASM433406v1	MCC10004	SAMN06368571	2.55173	60	52	2028
GCF_004334045.1_ASM433404v1	MCC10007	SAMN06368573	2.48463	60.2	59	2024
GCF_004334035.1_ASM433403v1	MCC10008	SAMN06368574	2.54367	60	80	2119
GCF_004334005.1_ASM433400v1	MCC10010	SAMN06368576	2.45245	60.4	93	1984
GCF_004333995.1_ASM433399v1	MCC10012	SAMN06368578	2.47987	59.6	67	1977
GCF_004333975.1_ASM433397v1	MCC10014	SAMN06368579	2.46621	60.2	59	2007
GCF_004333935.1_ASM433393v1	MCC10017	SAMN06368582	2.44883	59.7	60	1972
GCF_004333925.1_ASM433392v1	MCC10015	SAMN06368580	2.63029	59.9	83	2177
GCF_004333905.1_ASM433390v1	MCC10018	SAMN06368583	2.33041	60	32	1891
GCF_004333895.1_ASM433389v1	MCC10022	SAMN06368586	2.41904	59.7	55	1950
GCF_004333875.1_ASM433387v1	MCC10021	SAMN06368585	2.23575	60	58	1774
GCF_004333855.1_ASM433385v1	MCC10023	SAMN06368587	2.30816	60.3	41	1853
GCF_004333845.1_ASM433384v1	MCC10025	SAMN06368588	2.40884	60.1	56	1912
GCF_004333795.1_ASM433379v1	MCC10029	SAMN06368591	2.3461	59.9	55	1877
GCF_004333785.1_ASM433378v1	MCC10031	SAMN06368593	2.40115	60.1	41	1916
GCF_004333775.1_ASM433377v1	MCC10033	SAMN06368594	2.39204	60	63	1929
GCF_004333765.1_ASM433376v1	MCC10034	SAMN06368595	2.25363	60	70	1771
GCF_004333735.1_ASM433373v1	MCC10036	SAMN06368597	2.24998	59.9	22	1811
GCF_004333715.1_ASM433371v1	MCC10035	SAMN06368596	2.4575	59.8	52	2012
GCF_004333695.1_ASM433369v1	MCC10041	SAMN06368601	2.37064	60.1	47	1906
GCF_004333675.1_ASM433367v1	MCC10042	SAMN06368602	2.32056	60.1	53	1867
GCF_004333645.1_ASM433364v1	MCC10044	SAMN06368604	2.51281	60.3	50	2055
GCF_004333635.1_ASM433363v1	MCC10043	SAMN06368603	2.62386	59.5	62	2115
GCF_004333625.1_ASM433362v1	MCC10045	SAMN06368605	2.43778	60.3	61	1973
GCF_004333575.1_ASM433357v1	MCC10046	SAMN06368606	2.28641	59.9	71	1761
GCF_004333565.1_ASM433356v1	MCC10048	SAMN06368608	2.45602	59.8	71	1949
GCF_004333555.1_ASM433355v1	MCC10050	SAMN06368609	2.28037	59.8	34	1782
GCF_004333535.1_ASM433353v1	MCC10052	SAMN06368611	2.42874	60.1	65	1956
GCF_004333515.1_ASM433351v1	MCC10053	SAMN06368612	2.42582	60.3	51	1942
GCF_004333475.1_ASM433347v1	MCC10056	SAMN06368615	2.29975	60	79	1837
GCF_004333465.1_ASM433346v1	MCC10060	SAMN06368619	2.31311	60.2	59	1831
GCF_004333455.1_ASM433345v1	MCC10055	SAMN06368614	2.48603	60.1	72	2019
GCF_004333445.1_ASM433344v1	MCC10062	SAMN06368620	2.32592	59.8	59	1837
GCF_004333425.1_ASM433342v1	MCC10064	SAMN06368621	2.26578	60	40	1790
GCF_004333385.1_ASM433338v1	MCC10066	SAMN06368622	2.29383	59.8	53	1852
GCF_004333375.1_ASM433337v1	MCC10067	SAMN06368623	2.39437	59.6	55	1910
GCF_004333365.1_ASM433336v1	MCC10068	SAMN06368624	2.40787	59.7	58	1894
GCF_004333335.1_ASM433333v1	MCC10069	SAMN06368625	2.36718	59.9	57	1893
GCF_004333325.1_ASM433332v1	MCC10070	SAMN06368626	2.50822	59.6	48	2038
GCF_004333305.1_ASM433330v1	MCC10071	SAMN06368627	2.28772	60	49	1793
GCF_004333275.1_ASM433327v1	MCC10073	SAMN06368629	2.2843	59.7	42	1829
GCF_004333265.1_ASM433326v1	MCC10075	SAMN06368631	2.38497	60.1	53	1947
GCF_004333235.1_ASM433323v1	MCC10076	SAMN06368632	2.56355	60.2	50	2152
GCF_004333215.1_ASM433321v1	MCC10079	SAMN06368635	2.38272	59.9	77	1936
GCF_004333205.1_ASM433320v1	MCC10078	SAMN06368634	2.27198	59.8	58	1766
GCF_004333175.1_ASM433317v1	MCC10080	SAMN06368636	2.52893	60.2	59	2014
GCF_004333165.1_ASM433316v1	MCC10081	SAMN06368637	2.32346	59.9	56	1911
GCF_004333125.1_ASM433312v1	MCC10084	SAMN06368639	2.26435	60.1	46	1788
GCF_004333115.1_ASM433311v1	MCC10086	SAMN06368641	2.28382	59.8	48	1783
GCF_004333105.1_ASM433310v1	MCC10087	SAMN06368642	2.30319	60	49	1819
GCF_004333065.1_ASM433306v1	MCC10089	SAMN06368643	2.32481	60.3	41	1853
GCF_004333045.1_ASM433304v1	MCC10096	SAMN06368650	2.57129	59.7	39	2099
GCF_004333035.1_ASM433303v1	MCC10090	SAMN06368644	2.34731	59.8	49	1883
GCF_004333015.1_ASM433301v1	MCC10091	SAMN06368645	2.39421	60	64	1931
GCF_004333005.1_ASM433300v1	MCC10103	SAMN06368657	2.39468	59.9	16	1933
GCF_004332965.1_ASM433296v1	MCC10100	SAMN06368654	2.518	60.1	56	2037
GCF_004332945.1_ASM433294v1	MCC10116	SAMN06368666	2.62898	60	47	2167
GCF_004332935.1_ASM433293v1	MCC10112	SAMN06368662	2.27757	60	58	1804
GCF_004332925.1_ASM433292v1	MCC10092	SAMN06368646	2.23483	59.9	89	1742
GCF_004332895.1_ASM433289v1	MCC10094	SAMN06368648	2.50962	59.9	39	2078
GCF_004332865.1_ASM433286v1	MCC10093	SAMN06368647	2.45894	60.2	57	2000
GCF_004332855.1_ASM433285v1	MCC10095	SAMN06368649	2.35682	60.3	95	1915
GCF_004332835.1_ASM433283v1	MCC10098	SAMN06368652	2.32667	59.9	58	1815
GCF_004332825.1_ASM433282v1	MCC10097	SAMN06368651	2.28035	60	52	1805
GCF_004332765.1_ASM433276v1	MCC10107	SAMN06368659	2.38475	59.8	45	1931
GCF_004332755.1_ASM433275v1	MCC10102	SAMN06368656	2.53875	60.1	56	2077
GCF_004332745.1_ASM433274v1	MCC10099	SAMN06368653	2.34007	60.1	66	1870
GCF_004332735.1_ASM433273v1	MCC10106	SAMN06368658	2.41503	60.1	74	1947
GCF_004332725.1_ASM433272v1	MCC10101	SAMN06368655	2.41806	60.1	65	1953
GCF_004332665.1_ASM433266v1	MCC10108	SAMN06368660	2.41875	60.3	73	1972
GCF_004332655.1_ASM433265v1	MCC10111	SAMN06368661	2.43826	60	49	2007
GCF_004332645.1_ASM433264v1	MCC10115	SAMN06368665	2.43372	60.3	54	2017
GCF_004332635.1_ASM433263v1	MCC10113	SAMN06368663	2.46411	60	62	2021
GCF_004332625.1_ASM433262v1	MCC10114	SAMN06368664	2.45459	59.8	58	2005
GCF_002900845.1_ASM290084v1	CECT 7210	SAMEA3158508	2.4677	59.9	1	2009
GCF_002861445.1_ASM286144v1	UMB0788	SAMN08193649	2.45493	60.2	33	2051
GCF_002833285.1_ASM283328v1	APC1466	SAMN07958351	2.41998	59.8	51	1967
GCF_002833265.1_ASM283326v1	APC1476	SAMN07958355	2.53254	60	48	2094
GCF_002833255.1_ASM283325v1	DPC6320	SAMN07958364	2.33037	59.9	25	1807
GCF_002833215.1_ASM283321v1	DPC6323	SAMN07958366	2.39696	60.2	52	1911
GCF_002833205.1_ASM283320v1	APC1462	SAMN07958348	2.41778	60.3	27	1953
GCF_002833185.1_ASM283318v1	APC1464	SAMN07958349	2.34652	60.1	31	1873
GCF_002833175.1_ASM283317v1	APC1465	SAMN07958350	2.45221	59.7	57	1976
GCF_002833135.1_ASM283313v1	APC1468	SAMN07958352	2.39516	60.2	45	1966
GCF_002833125.1_ASM283312v1	APC1473	SAMN07958354	2.31707	59.8	39	1817
GCF_002833115.1_ASM283311v1	APC1472	SAMN07958353	2.36404	60.2	50	1863
GCF_002833075.1_ASM283307v1	APC1477	SAMN07958356	2.22881	59.8	24	1726
GCF_002833065.1_ASM283306v1	APC1480	SAMN07958358	2.47775	59.9	27	2022
GCF_002833055.1_ASM283305v1	APC1478	SAMN07958357	2.22335	59.8	21	1729
GCF_002833035.1_ASM283303v1	APC1482	SAMN07958359	2.33744	60.2	72	1858
GCF_002833015.1_ASM283301v1	DPC6316	SAMN07958362	2.39397	60.4	32	1912
GCF_002832995.1_ASM283299v1	DPC6321	SAMN07958365	2.38236	59.9	28	1894
GCF_002832985.1_ASM283298v1	APC1503	SAMN07958360	2.5627	59.7	39	2103
GCF_002832955.1_ASM283295v1	APC1504	SAMN07958361	2.31029	60.2	51	1860
GCF_002832945.1_ASM283294v1	DPC6317	SAMN07958363	2.44863	60.2	20	1918
GCF_002276185.1_ASM227618v1	Indica	SAMN07503177	2.37423	60	43	1948
GCF_002076095.1_Bbif1886B	1886B	SAMN06621706	2.47375	60.2	47	2083
GCF_002076015.1_Bbif1890B	1890B	SAMN06621710	2.34167	59.9	109	1846
GCF_002075875.1_Bbif1898B	1898B	SAMN06621716	2.47439	59.9	41	1998
GCF_001940535.1_BlonW11v1	W11	SAMN06109230	2.32998	59.9	22	1857
GCF_001892965.1_ASM189296v1	296B	SAMN05916052	2.25318	59.9	40	1685
GCF_001725985.1_ASM172598v1	AH1206	SAMN04576213	2.42129	60.2	1	1967
GCF_001719085.1_ASM171908v1	35624	SAMN04254466	2.26406	60	1	1773
GCF_001686245.1_ASM168624v1	LO-K29b	SAMD00047623	2.37271	60.1	97	1866
GCF_001686225.1_ASM168622v1	LO-K29a	SAMD00047622	2.44918	60	85	1874
GCF_001686205.1_ASM168620v1	LO-C29	SAMD00047621	2.48387	60	49	1927
GCF_001686185.1_ASM168618v1	LO-21	SAMD00047620	2.65603	60.1	71	2034
GCF_001686165.1_ASM168616v1	LO-10	SAMD00047619	2.54024	60.3	80	1988
GCF_001686145.1_ASM168614v1	LO-06	SAMD00047618	2.43747	60	77	1926
GCF_001595465.1_ASM159546v1	379	SAMN04155602	2.38762	60.2	24	1921
GCF_001546275.1_ASM154627v1	CMW7750	SAMN03842222	2.37208	60	39	1894
GCF_001516925.1_ASM151692v1	MC-42	SAMN04263942	2.28825	59.8	29	1792
GCF_001447975.1_ASM144797v1	7	SAMN04129533	2.23558	60	36	1766
GCF_001447955.1_ASM144795v1	9	SAMN04129541	2.23377	60	31	1765
GCF_001446275.1_ASM144627v1	CCUG30698	SAMN03785819	2.458	60.2	1	1956
GCF_001446255.1_ASM144625v1	NCIMB8809	SAMN03785818	2.34099	60.1	1	1807
GCF_001293145.1_ASM129314v1	BG7	SAMN03271682	2.45576	60.0068	2	1926
GCF_001275745.1_assBLOI2	BLOI2	SAMN03775040	2.41759	60	72	1937
GCF_001051015.2_ASM105101v2	CECT 7210	SAMEA3158508	2.4677	59.9	1	1992
GCF_001050555.1_ASM105055v1	CECT 7347	SAMEA3146249	2.32722	60	128	1868
GCF_000829295.1_ASM82929v1	105-A	SAMD00019943	2.29014	60.1	1	1772
GCF_000786175.1_ASM78617v1	VMKB44	SAMN03105207	2.50193	60.3	34	2080
GCF_000772485.1_ASM77248v1	GT15	SAMN03093230	2.33752	60	1	1815
GCF_000741245.1_Biflon_sub.lon	LMG 13197	SAMN02673437	2.3847	60.3	8	1803
GCF_000730135.1_ASM73013v1	EK13	SAMN02862997	2.47453	60	39	2043
GCF_000730105.1_ASM73010v1	1-5B	SAMN02862991	2.36751	60.1	25	1902
GCF_000730055.1_ASM73005v1	7-1B	SAMN02862992	2.40709	59.8	34	1904
GCF_000730045.1_ASM73004v1	72B	SAMN02862994	2.37445	60.3	30	1950
GCF_000730035.1_ASM73003v1	17-1B	SAMN02862993	2.4672	60.2	20	1962
GCF_000730025.1_ASM73002v1	EK5	SAMN02862996	2.23129	59.7	28	1780
GCF_000497735.1_BLONGv1.0	E18	SAMN02471972	2.37297	60	1	1912
GCF_000478525.1_blongD2957	D2957	SAMN02472064	2.33023	60.4	13	1812
GCF_000261265.1_Blongum44Bv1.0	44B	SAMN00829148	2.55922	59.7	62	2109
GCF_000261245.1_Blongum16Bv1.0	1-6B	SAMN00829154	2.68677	59.6	171	2215
GCF_000261225.1_Blongum35Bv1.0	35B	SAMN00829158	2.51443	60.1	131	1967
GCF_000261205.1_Blongum22Bv1.0	2-2B	SAMN00829155	2.6257	59.7	141	2089
GCF_000219455.1_ASM21945v1	KACC 91563	SAMN02603656	2.39576	59.8115	3	1856
GCF_000210755.1_ASM21075v1	F8	SAMEA3138379	2.38499	59.9	1	1884
GCF_000196575.1_ASM19657v1	157F	SAMD00060953	2.40883	60.111	3	1923
GCF_000196555.1_ASM19655v1	JCM 1217	SAMD00060951	2.38516	60.3	1	1870
GCF_000185665.1_ASM18566v1	12_1_47BFAA	SAMN02463822	2.40599	60.1	61	1981
GCF_000166895.2_ASM16689v2	DJO10A	SAMN02441414	2.37528	59.9	120	1792
GCF_000166315.1_ASM16631v1	BBMN68	SAMN02603469	2.26594	59.9	1	1740
GCF_000155415.1_ASM15541v1	CCUG 52486	SAMN02463677	2.48085	60	22	2034
GCF_000008945.1_ASM894v1	DJO10A	SAMN02603512	2.38953	60.1182	3	1932
GCF_000007525.1_ASM752v1	NCC2705	SAMN02603675	2.26027	60.1075	2	1773
GCF_000003135.1_ASM313v1	ATCC 55813	SAMN00001475	2.39636	60.1	114	1901
GCF_003094635.1_ASM309463v1	DS9_3	SAMN06464100	2.39717	59.9	13	1955
GCF_003094855.1_ASM309485v1	DS15_3	SAMN06464097	2.39818	59.9	21	1956
GCF_003094935.1_ASM309493v1	DS18_3	SAMN06464098	2.44826	59.7	174	2002
GCF_003094955.1_ASM309495v1	DS1_3	SAMN06464096	2.41728	60	170	1958
GCF_003094975.1_ASM309497v1	DS7_3	SAMN06464099	2.23729	60	17	1765
GCF_003094995.1_ASM309499v1	DS32_3	SAMN08949007	2.23593	60.1	28	1761

**Table 3:** Parameters for self-sequenced genomes.

	Scaffold Number	Length	Gap	Average Length	N50	N90	GC Content (%)
FGSZY6M4	52	2,321,455	3574	44,643.37	202,550	83,023	59.72
RG4-1	51	2,601,515	3420	51,010.1	224,480	60,103	60.21
M1-20-R01-3	46	2,237,922	3258	48,650.48	232,217	63,500	60.02

**Table 4:** Strain-specific genes identified by Roary for *B. longum* RG4-1.

Gene	Non-Unique Gene Name	Annotation	Avg Group Size Nuc	Gene Tag
group_8150		hypothetical protein	227	RG4-1_00079
group_8151		hypothetical protein	296	RG4-1_00112
group_8152		hypothetical protein	257	RG4-1_00222
group_8153	xerC_1	Tyrosine recombinase XerC	1286	RG4-1_00224
group_8154		Helix-turn-helix domain protein	185	RG4-1_00225
group_8155		Helix-turn-helix domain protein	395	RG4-1_00226
group_8156		Helix-turn-helix domain protein	341	RG4-1_00227
group_8157		site-specific tyrosine recombinase XerC	1349	RG4-1_00228
group_8158		hypothetical protein	209	RG4-1_00545
group_8160		hypothetical protein	236	RG4-1_00568
group_8161		hypothetical protein	272	RG4-1_01044
group_8162	mdeA_1	Methionine gamma-lyase	1277	RG4-1_01045
group_8163		Phage-related minor tail protein	3317	RG4-1_01165
group_8164		Phage tail protein	788	RG4-1_01166
group_8165		hypothetical protein	1115	RG4-1_01167
group_8166	smc_4	Chromosome partition protein Smc	1124	RG4-1_01168
group_8167		hypothetical protein	359	RG4-1_01169
group_8168		hypothetical protein	1871	RG4-1_01170
group_8169		hypothetical protein	185	RG4-1_01171
group_8170	acm	Lysozyme M1 precursor	1280	RG4-1_01174
yoaD		Putative 2-hydroxyacid dehydrogenase YoaD	968	RG4-1_01873
group_8172	araN_4	putative arabinose-binding protein precursor	1331	RG4-1_01874
ycjP_2		Inner membrane ABC transporter permease protein YcjP	830	RG4-1_01875
group_8174	ycjO_1	Inner membrane ABC transporter permease protein YcjO	899	RG4-1_01876
group_8175		hypothetical protein	161	RG4-1_01877
group_8176	nanE	Putative N-acetylmannosamine-6-phosphate 2-epimerase	689	RG4-1_01878
group_8177	nanA	N-acetylneuraminate lyase	917	RG4-1_01879
bglK		Beta-glucoside kinase	914	RG4-1_01880
rpiR		HTH-type transcriptional regulator RpiR	905	RG4-1_01881
group_8180		Chitinase class I	551	RG4-1_02208
group_8181		Thaumatin family protein	284	RG4-1_02209
group_8182		hypothetical protein	197	RG4-1_02210

**Table 5:** Strain-specific genes identified by Roary for *B. longum* M1-20-R01-3.

Gene	Non-Unique Gene Name	Annotation	Avg Group Size Nuc	Gene Tag
group_6841		hypothetical protein	194	M1-20-R01-3_00305
group_6842		hypothetical protein	998	M1-20-R01-3_00310
group_6843		hypothetical protein	221	M1-20-R01-3_00311
group_6844		hypothetical protein	233	M1-20-R01-3_00316
group_6845		hypothetical protein	257	M1-20-R01-3_00318
group_6846		hypothetical protein	623	M1-20-R01-3_00319
group_6847		hypothetical protein	587	M1-20-R01-3_00320
group_6848		hypothetical protein	1745	M1-20-R01-3_00324
group_6849		hypothetical protein	236	M1-20-R01-3_00325
group_6850		hypothetical protein	581	M1-20-R01-3_00326
group_6851		hypothetical protein	191	M1-20-R01-3_00327
group_6852		YcfA-like protein	224	M1-20-R01-3_00328
group_6853		hypothetical protein	413	M1-20-R01-3_00329
group_6854		hypothetical protein	617	M1-20-R01-3_00562

**Table 6:** Strain-specific genes identified by Roary for *B. longum* FGSZY6M4.

Gene	Non-Unique Gene Name	Annotation	Avg Group Size Nuc	Gene Tag
group_3844		hypothetical protein	203	FGSZY6M4_00001
group_3845	pepD_1	Dipeptidase	1637	FGSZY6M4_00002
group_3846	epsH	Putative glycosyltransferase EpsH	1034	FGSZY6M4_00003
group_3847		transcriptional regulator BetI	821	FGSZY6M4_00004
group_3850		N-acetylmuramoyl-L-alanine amidase	878	FGSZY6M4_00052
group_3869		hypothetical protein	419	FGSZY6M4_00335
group_3870		putative ABC transporter ATP-binding protein/MT1014	434	FGSZY6M4_00336
zur_2		Zinc uptake regulation protein	500	FGSZY6M4_00339
group_3880		hypothetical protein	578	FGSZY6M4_00378
group_3899		hypothetical protein	518	FGSZY6M4_00692
yhcR_2		Endonuclease YhcR precursor	3566	FGSZY6M4_01406
group_3960		hypothetical protein	332	FGSZY6M4_01466
group_3961		hypothetical protein	692	FGSZY6M4_01467
group_3962		YcaO-like family protein	1610	FGSZY6M4_01468
group_3963		ABC-2 type transporter	731	FGSZY6M4_01469
group_3964	yxlF	putative ABC transporter ATP-binding protein YxlF	908	FGSZY6M4_01470
group_3965		hypothetical protein	188	FGSZY6M4_01471
group_3966		hypothetical protein	1079	FGSZY6M4_01472
group_3967		hypothetical protein	1061	FGSZY6M4_01473
group_3968		hypothetical protein	2594	FGSZY6M4_01474
group_3969		Nitroreductase family protein	1532	FGSZY6M4_01475
group_3970		YcaO-like family protein	1607	FGSZY6M4_01476
group_3971		hypothetical protein	1691	FGSZY6M4_01477
group_3972		hypothetical protein	176	FGSZY6M4_01478
group_4002		hypothetical protein	254	FGSZY6M4_01825
group_4004		hypothetical protein	308	FGSZY6M4_01832
group_4005		hypothetical protein	227	FGSZY6M4_01836
group_4008		hypothetical protein	365	FGSZY6M4_01863
group_4009		hypothetical protein	359	FGSZY6M4_01864
group_4010		hypothetical protein	197	FGSZY6M4_01866
group_4011		hypothetical protein	455	FGSZY6M4_01867
group_4012	whiB1_2	Transcriptional regulator WhiB1	245	FGSZY6M4_01868
group_4013		hypothetical protein	329	FGSZY6M4_01869
group_4014		hypothetical protein	170	FGSZY6M4_01871
group_4036		hypothetical protein	4835	FGSZY6M4_01918
group_4037		hypothetical protein	566	FGSZY6M4_01919
group_4038		hypothetical protein	440	FGSZY6M4_01920
group_4039		hypothetical protein	176	FGSZY6M4_01921
group_4040		hypothetical protein	527	FGSZY6M4_01922
group_4041		hypothetical protein	368	FGSZY6M4_01923
group_4042		hypothetical protein	566	FGSZY6M4_01939
group_4048		hypothetical protein	338	FGSZY6M4_01977
group_4049		hypothetical protein	419	FGSZY6M4_01981
group_4050		hypothetical protein	1190	FGSZY6M4_01982
bvgA		Virulence factors putative positive transcription regulator BvgA	668	FGSZY6M4_01983
group_4052		enterobactin exporter EntS	1262	FGSZY6M4_01984
group_4053		hypothetical protein	197	FGSZY6M4_01985
aacA-aphD		Bifunctional AAC/APH	1340	FGSZY6M4_01986
group_4055		Zein seed storage protein	755	FGSZY6M4_01993

**Table 7:** Strain-specific primers for three *B. longum* strains.

Strain	Primer Sequence (5′-3′)	Primer Length (bp)	Primer Score	Product Length (bp)
RG4-1	F: ACCATCTGGGTGGAGAAAGTG	21	100	115
	R: TGGCGGAAATGAACTCGTAAT	21	100	
M1-20-R01-3	F: GATGGCACCAGCACAGG	17	100	199
	R: GGAGCACGGCGACTATG	17	100	
FGSZY6M4	F: TCCCGAATCCGACTATGA	18	100	144
	R: TCGCTGCCAACTACTAAAA	19	100	

## Data Availability

Data in this study are available from the authors upon request.
